# Exploring the recruitment, ethical considerations, conduct and information dissemination of an audiology trial: a pretrial qualitative study (q-COACH)

**DOI:** 10.1186/s13063-019-3968-1

**Published:** 2020-01-06

**Authors:** Emilie Francis-Auton, Chris Warren, Jeffrey Braithwaite, Frances Rapport

**Affiliations:** 1 Australian Institute of Health Innovation, Faculty of Medicine and Health Sciences, Macquarie University, Level 6, 75 Talavera Rd, Sydney, NSW 2109 Australia; 2 Cochlear Ltd, 1 University Ave, Macquarie Park, NSW 2113 Australia

**Keywords:** Randomised controlled trials, Audiology, Qualitative research, Hearing devices, Cochlear implants, Hearing loss

## Abstract

**Introduction:**

Randomised controlled trials (RCTs), while still considered the gold standard approach in medical research, can encounter impediments to their successful conduct and the dissemination of results. Pretrial qualitative research can usefully address some of these impediments, including recruitment and retention, ethical conduct, and preferred methods of dissemination. However, pretrial qualitative work is rarely undertaken in audiology. The Comparison of outcomes with hearing aids and cochlear implants in adults with moderately severe-to-profound bilateral sensorineural hearing loss (COACH) is a proposed RCT aiming to clarify when hearing aids (HAs) or cochlear implants (CIs) are the most suitable for different degrees of hearing loss and for which kinds of patients. q-COACH is a pretrial, qualitative study examining stakeholders’ experiences of HAs and CIs, current clinical practices and stakeholders’ perspectives of the design, conduct and dissemination plans for the proposed COACH study.

**Methods:**

Twenty-four participants including general practitioners, audiologists, adult HA users, and adult support networks undertook either semi-structured individual or paired interviews and completed demographic questionnaires. Data were analysed thematically.

**Results:**

Four key themes arose from this study: 1) rethinking sampling and recruitment strategies, 2) ethical considerations, 3) refining trial conduct, and 4) interconnected, appropriate and accessible methods of results dissemination.

**Conclusions:**

This qualitative investigation identified key considerations for the proposed RCT design, conduct and dissemination to help with successful implementation of COACH, and to indicate a plan of action at all RCT stages that would be acceptable to potential participants. By drawing on the perspectives of multiple key stakeholders and including a more general discussion of their experience and opinions of hearing loss, hearing device use and service availability, the study revealed experiential and ethical paradigms in which stakeholders operate. In so doing, q-COACH has exposed the benefits of preliminary qualitative investigations that enable detailed and rich understandings of the phenomenon at stake, forestalling problems and improving the quality of trial design, conduct and dissemination, while informing future RCT development discussions.

## Background

Hearing loss is a significant disabling issue which, when left untreated, has been linked to social isolation and loneliness [1, 2], depression [3, 4], falls [5], and cognitive impairment and dementia [6, 7]. The uptake of hearing aids (HAs) and cochlear implants (CIs) for the treatment of hearing-impaired individuals is low, with multiple sources reporting that these devices remain underutilised. The prevalence of HA use amongst those with hearing loss ranges from 14.2% to 33.1% [8–10] and the uptake of CIs is 10% or less of the people who clinically need them in adult populations globally [11]. The well-documented benefits of hearing devices include improved hearing ability, improved sound quality, and enhanced quality of life [12–15]. One important barrier to uptake is the paucity of high-quality, accessible, clinical evidence of the viability or effectiveness of HAs compared to CIs.

This dearth of information has created clinical equipoise [16]; there is a lack of certainty amongst healthcare professionals (HCPs) about appropriate decision-making processes, in this case regarding the relative merits of HAs and CIs. Clinical equipoise is reinforced by personal preference and personal decision-making. HCPs may, for example, lack awareness of the benefits of CIs for patients with significant hearing loss which affects their assessment and referral for implantation, and limits informative discussions with patients [11, 17–19]. Decision-making processes can also be influenced by the commercial interests of hearing clinics where sales commissions and targets motivate some audiologists in their prescribing habits [20, 21]. These sales techniques, often directed at vulnerable or disadvantaged populations (due to factors such as hearing loss, age, other medical conditions, and income), can lead to patients purchasing costly or unnecessary HAs [20, 21]. Whether considering HAs or CIs, it should be possible for HCPs and patients to reach a balanced, informed decision on the most appropriate treatment choice on all occasions. For this to occur, HCPs and patients need high-quality information about all hearing devices. A randomised controlled trial (RCT) would offer a high-quality, generalizable information source, and could evaluate the outcomes and cost effectiveness of HAs and CIs and their comparative qualities, as well as the facilitators and barriers to their uptake.

When well designed and implemented, RCTs provide methodologically robust ways of preparing interventions for assessment and producing ‘best evidence’ to inform clinical decision-making. The value of qualitative research in relation to RCTs is well recognised and utilised in the health field [22]; however, it is rarely used in audiology [23]. Qualitative research adds to our understanding of people’s complex social worlds, asking questions about behaviour, relationships and lived experiences. Qualitative research can also address RCT recruitment, trial delivery, the dissemination of trial results, and provide recommendations for future trials [24]. Qualitative research can be used at all stages of an RCT, although pretrial research, which is currently underutilised, has the potential to optimise trial design, conduct and methods of dissemination for both current and future studies [24].

The qualitative Comparison of outcomes with hearing aids and cochlear implants in adults with moderately severe-to-profound bilateral sensorineural hearing loss study (q-COACH) had the aim of exploring stakeholders’ experiences of device provision or use, and their perspectives of the proposed Comparison of outcomes with hearing aids and cochlear implants in adults with moderately severe-to-profound bilateral sensorineural hearing loss (COACH) trial in terms of its planned design, conduct and dissemination methods. q-COACH sought to provide recommendations that might be used to refine and improve the recruitment procedures, the ethical parameters, the delivery and the dissemination methods of COACH, and to offer insights which could be used to inform future audiology trials. The objectives were to ensure a more appropriate RCT design that could meet the needs of patients, support persons and HCPs, as well as to identify the best methods for information dissemination. The utility of such an approach is recognised through the trial literature [25], not only to inform specific stages of a trial, but also to embed suitable information across trial methodology.

## Methods

### The research setting

**Fig. 1:**
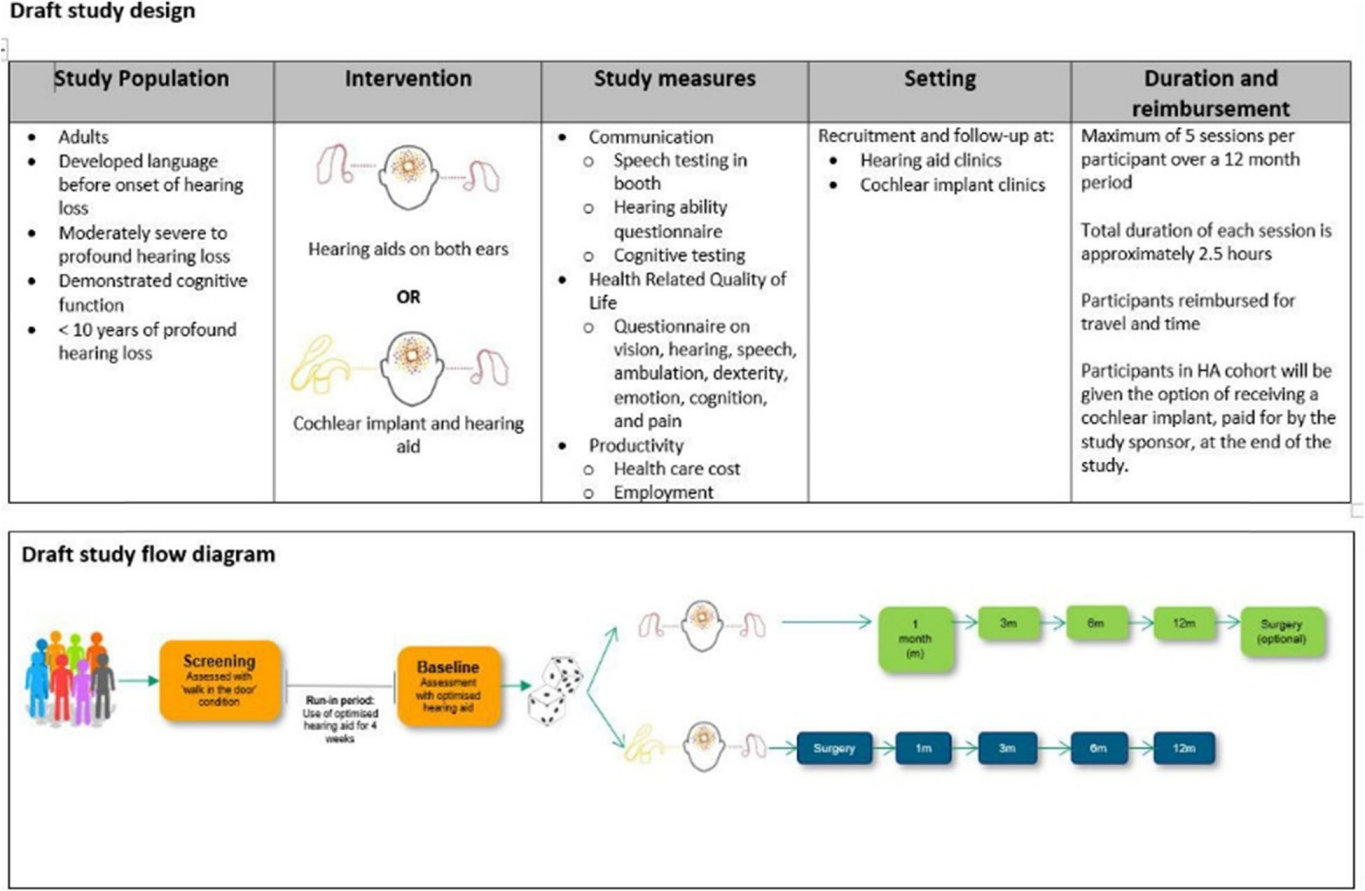
Draft study design and flow diagram: the COACH study

The proposed RCT (COACH) aims to recruit participants 18 years of age and over with moderately severe-to-profound hearing loss (≥56 dB hearing loss mean pure tone threshold) [26] for a period of no more than 10 years. Participants will be initially screened and then randomly allocated to one of two trial arms: 1) those undergoing cochlear implantation in one ear and fitted with an HA in the other; and 2) those fitted with two HAs and a 4-week acclimatisation period. All participants will attend a 2.5-h assessment session, with five sessions in total over a 12-month period. Each session requires participants to undertake several study measures including communication tests (for example, hearing tests), health-related quality of life questionnaires and productivity questionnaires. Participants will be reimbursed for their time and travel. The main form of reimbursement will be a CI manufactured by Cochlear Ltd., with those randomly allocated to the CI trial arm being implanted at the start of the RCT and those within the HA trial arm being implanted at the conclusion of the RCT. The proposed COACH study has been designed and will be commissioned by Cochlear Ltd. (see Fig. 1).

### Qualitative study design

This qualitative study (q-COACH) aimed to: 1) identify stakeholders’ experiences of, and insights into, hearing loss and hearing loss devices; 2) assess how the COACH study should be designed, conducted and reported and for whose benefit; 3) clarify how information generated from the COACH study could be best disseminated and with which stakeholder groups; and 4) consider how outcomes from dissemination might be sustained (see the published study protocol for more detail [27]). Ethical approval for this project was obtained from the Macquarie University Human Research Ethics Committee (approval number 5201833514848).

q-COACH involved individual or paired interviews, while all participants were also expected to complete a demographic questionnaire. Interviews ensured that meanings attributed to experience were participant- rather than researcher-driven [28]. The flexible nature of interviews allowed unexpected but significant issues to arise and enabled a more in-depth exploration of an idea or response [28]. Paired interviews, a relatively underutilised technique [22, 29, 30], were necessary when it was the preferred option for the participant and their partner (where participants had a relationship or connection with one another). They helped participants feel more comfortable in the interview scenario, provided opportunities for considered responses (with paired partners building on or challenging each other’s narratives) [31], and offered an additional level of emotional support.

### Sample and recruitment

Participants were recruited using time-frame sampling and snowball sampling in combination. Time-frame sampling removes opportunistic recruitment of participants as it is dependent not on the person but on the time frame, with all eligible participants given an equal opportunity of recruitment over a certain period, thereby reducing selection bias. To ensure data saturation was achieved within the predefined time frame, time-frame sampling was coupled with snowball sampling strategies (where an eligible participant then recommends others they know who fit the eligibility criteria and who can be approached) [28] for all four cohorts. We aimed to recruit participants who would be eligible for the proposed RCT (in other words, who had moderately severe-to-profound hearing loss for <10 years).

The team was able to build on established recruitment strategies and principles of which they had previous experience in the audiology field [32, 33], producing multiple, consistent recruitment pathways. Promotional flyers were sent to general practitioner (GP) surgeries and clinics via professional network e-newsletters, audiology clinics, hearing associations and posts on social media websites (such as Facebook). The study field researcher (EF-A) also sat in on an Australian university-based speech and hearing clinic where study flyers were displayed and where potential participants (audiologists, HA users, and members of their support network) could ask questions about the study.

### Data collection and analysis

Twenty-four participants undertook either individual semi-structured or paired interviews and completed demographic questionnaires (see Table 1). The participants were split across four cohorts: five GPs, nine HA and CI audiologists, nine HA users, and five members of HA users’ support networks (some participants identified as being a part of two cohorts). In-depth interviews allowed for the exploration of participant knowledge and experience, opinion and perception of HAs and CIs, on hearing device use and fitting/implant, clinical care and referral pathways and questions were posed about the proposed COACH study design, conduct and dissemination methods (Table 2).

**Table 1:** Demographic data from patients, support persons and HCPs

	HA users (*n* = 9)	SPs (*n* = 5)	GPs (*n* = 5)	Audiologists (*n* = 9)
Age				
18–35 years	0	0	0	2
35–50 years	3	1	1	7
50–64 years	1	1	4	0
65–74 years	1	1	0	0
75 years or older	3	1	0	0
Prefer not to answer	1	0	0	0
Gender				
Male	3	1	2	2
Female	6	3	3	7
Had private health insurance				
Yes	5	N/A	N/A	N/A
No	2	N/A	N/A	N/A
Prefer not to answer	2	N/A	N/A	N/A
State of residence or (for HCP) workplace				
New South Wales	7	4	5	9
South Australia	1	0	0	0
Queensland	1	0	0	0
Urban/rural location of residence or (for HCP) workplace				
Metropolitan resident	6	2	4	9
Regional/rural residents	3	2	1	0

*GP* general practitioner, *HA* hearing aid user, *HCP* healthcare professional, *N/A* not applicable, *SP* support person

**Table 2:** Summary of interview topics

Topics for patient and support person interviews	Topics for HCP (audiologist and GP) interviews
• Experience of hearing loss, hearing devices, and health services associated with hearing loss	• Professional experience in relation to hearing loss, hearing devices, referral pathways
• Experiences of information dissemination for hearing devices	• Current sources of information on hearing devices
• Prior knowledge of hearing loss devices (distribute Fig. 2)	• Prior knowledge of hearing loss devices (distribute Fig. 2)
• Understanding of trial information (distribute Fig. 1)	• Understanding of trial information (distribute Fig. 1)
• Views and suggestions on trial recruitment	• Views and suggestions on trial recruitment
• Views and suggestions on conduct of study (including factors influencing potential participation in COACH; randomisation; retention; appropriateness, frequency and duration of study measures; reimbursement)	• Views and suggestions on conduct of study (including factors influencing potential participation in COACH; randomisation; retention; appropriateness, frequency and duration of study measures; reimbursement)
• Potential ethical issues and suggestions for resolutions	• Potential ethical issues and suggestions for resolutions
• Importance and impact of research on HCP–patient relationships and referral pathways	• Importance and impact of research on HCP–patient relationships and referral pathways
• Preferred pathways for dissemination of COACH results	• Preferred pathways for dissemination of COACH results

*COACH* Comparison of outcomes with hearing aids and cochlear implants in adults with moderately severe-to-profound bilateral sensorineural hearing loss, *GP* general practitioner, *HCP* healthcare professional

**Fig. 2:**
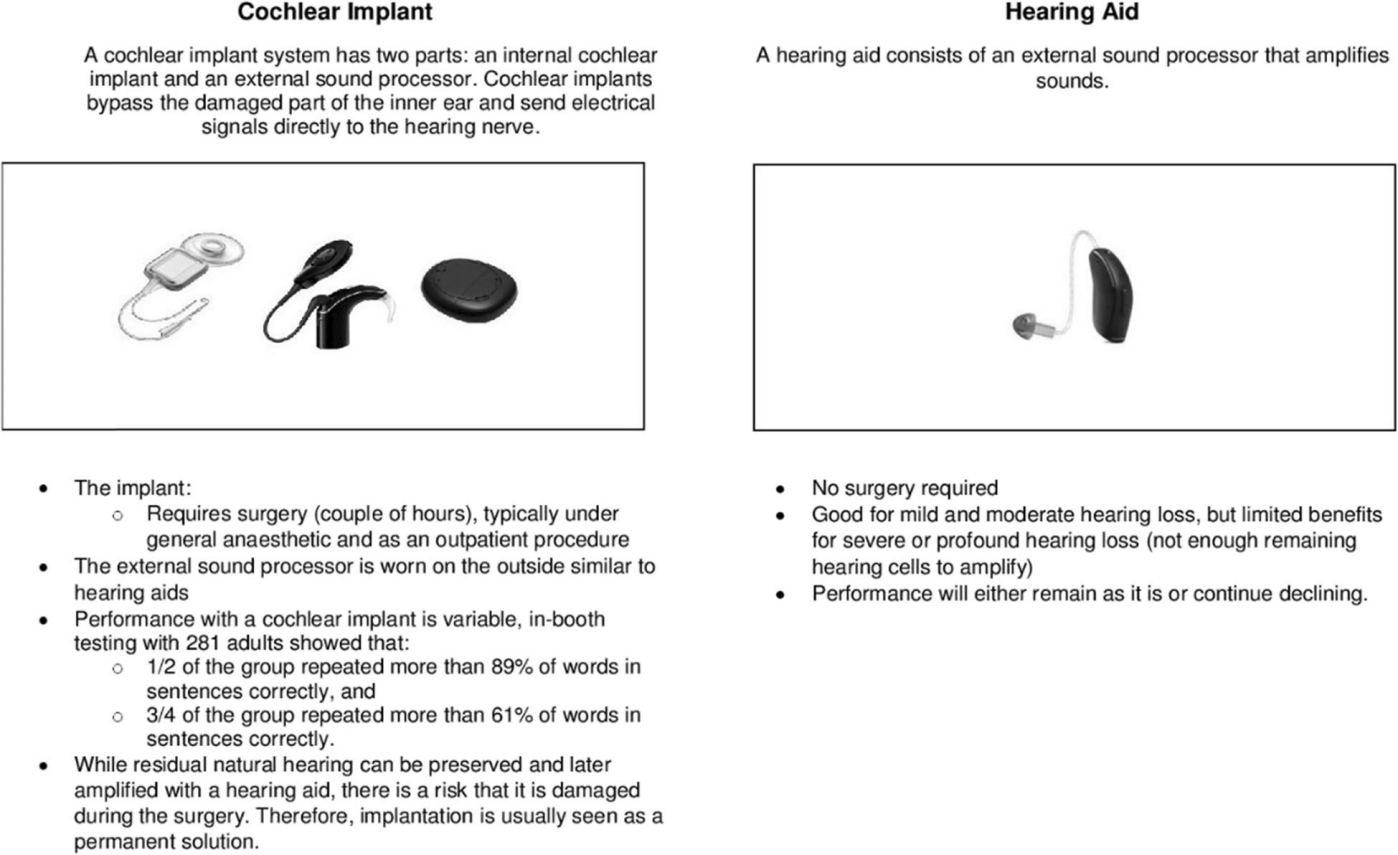
Comparing hearing aids and cochlear implants

During the interviews, participants were shown a chart which compared HAs and CIs (Fig. 2) and a draft of the proposed COACH study (Fig. 1) in order to facilitate discussion. Participants undertook either face-to-face individual interviews (*n* = 11), face-to-face paired interviews (*n* = 6), individual telephone interviews (*n* = 3), paired telephone interviews (*n* = 2) or individual interviews via email correspondence (*n* = 2), depending on their preference. All participants who contacted the researcher for further details participated in the study. Interviews were predominantly undertaken in the audiology clinic’s private rooms. We encouraged participants to undertake face-to-face interviews; however, based on our previous qualitative research in audiology [33], we decided to include multiple modes of interviewing to ensure participants were comfortable and to allow those who were time-poor to participate (such as GPs) and to allow for interviews to take place with participants who reside outside New South Wales but within Australia.

Interviews were conducted by EF-A who is an experienced qualitative researcher with a PhD in sociology. It is important to note that EF-A had no prior relationship with participants. Interviews lasted 30 min to 1 h and took place between November 2018 and April 2019. Informed written consent was obtained from all participants. Where possible, EF-A made notes about interview participant dynamics including body language, communication approaches and gestures to add rich detail to the textual data capture. EF-A found it easiest to establish rapport in the face-to-face interviews which led to high-quality interviews. The depth of the data generated via email and telephone interviews was, however, still satisfactory. Data saturation was achieved across the four stakeholder groups (assessed when no new themes were in evidence within the data being collected).

For the interviews with participants with hearing loss, the study researcher adopted multiple strategies to ensure that communication was clear and comprehensive. This included quiet rooms, maintaining face-to-face contact during interviews, examining any nonverbal signs that participants had not understood the information, and providing an interview guide for each participant to support the verbal delivery of questions (note that the interviews were semi-structured and therefore not all questions asked appeared on the interview guide). Participants were offered a stipend as a gesture of appreciation for any travel involved and for their time during data collection.

Interviews were audiorecorded, transcribed verbatim, and uploaded to NVivo 12 software.

Two experienced qualitative researchers (EF-A and FR) undertook thematic analysis [34] of the datasets; EF-A examined the entire dataset while FR examined a portion of the dataset as is common practice in qualitative research. The first stage of analysis involved repeated readings of each transcript, noting down ideas and impressions, searching for patterns and ensuring while this was happening that the researchers maintained the complexity of the accounts, while noting any negative cases across the dataset. We independently identified themes and then discussed these together. Using constant comparison, we searched through interviews to compare, contrast and eventually refine themes. Finally, we drew on literature to find useful analytic concepts to make meaning of the patterns we identified in the data. All analysis was in keeping with the study aims and objectives.

## Results

Results are reported in a narrative style according to the key thematic findings. All themes include verbatim quotations, labelled according to who was speaking and the mode of interview (i.e. ‘A’ represents audiologists, ‘HA’ represents hearing aid users, ‘SP’ represents support person, ‘GP’ represents general practitioner, ‘F’ represents face-to-face, ‘I’ represents individual, ‘P’ represents paired, ‘T’ represents telephone and ‘E’ represents email). When two of these labels are used (for example ‘HA5-GP2’) this means that the participant identified as belonging to two stakeholder groups (such as an HA user and a GP).

### Rethinking sampling and recruitment strategies

#### Significant and static barriers to recruitment

HCPs largely agreed that recruitment to the COACH study would be difficult, and participation would be dependent on the potential participants’ attitudes to HAs and CIs as regards initial interest. As participation in the COACH study entails CI surgery, HCPs reflected that recruitment barriers would mirror general barriers to CIs. These barriers include fear of surgery, people considering themselves too old for surgery, concerns that surgery is irreversible, fear of losing residual hearing, cosmetic reasons, belief that their quality of life would not improve markedly and that HAs are adequate for everyday activities. Based on their professional experiences, HCPs commented that the transition from HAs to CIs is significant and lengthy and therefore these barriers are not easy to overcome. Indeed, HCPs stated that in some cases it would not be ethical or time-effective to attempt to circumvent these barriers.

Two patients were interested in an implant but didn’t go ahead with it … because [one patient] was very scared of surgery and the potential of losing all residual hearing. The other patient didn’t go ahead with it because he thought that he was just a bit older and didn’t know if it was worth it, even though it was explained that age is not a limitation. (A8-T-P)

From my own, you know, experiences, the leap that people make from a hearing aid to a cochlear implant is a huge one, and often one that does take a fair amount of time and encouragement and sometimes, over many appointments, you know, like, you know, over a year or a few years … I think this is going to be your hardest, recruitment. (A6-F-I)

We'll sometimes we have in our notes, ‘mentioned cochlear implant before, client adamant, does not want, do not mention again’ because it is a point of sometimes massive contention, you know, that they have made this decision, it is their decision to make, they don't want to be hounded. So, you kind of need to target people who've already been thinking about it … Those people that have already done a little bit of research and had it presented to them as an idea. (A2-F-I)

Consistent with HCP concerns over recruitment, most HA users, in particular those who had already decided against cochlear implantation, confirmed that they would not participate in the COACH study. The reasons provided were consistent with the reasons specified by HCPs.

Chopping into the bones and stuff. No thank you … If it’s for a very deaf child and going to change their life, wonderful. If you’re 87 and don’t want your skull fiddled with — no thank you. (HA1-F-I)

I wouldn’t consider a cochlear implant because I do know that there is a slight risk that it wouldn’t be successful. If that’s the case the nerves are damaged for your hearing and you won’t go back to what you had. Is that right? (HA4-F-I)

#### Targeted recruitment

As a result of these recruitment barriers, HCPs recommended targeting multiple recruitment sites where the RCT researchers could access potential participants who are receptive to the idea of CIs but have not yet embarked on the surgery. Moreover, targeting participants at this stage would ensure that they are more likely to be psychologically and physically prepared to embark on cochlear implantation. Indeed, the HA users (three out of nine) who expressed interest in CIs confirmed that they would participate in the COACH study. A diverse portfolio of recruitment sites within Australia were recommended, including: the Sydney Cochlear Implant Centre, audiology clinics, ear, nose and throat (ENT) surgeons’ practices, Better Hearing Australia, Australian Hearing, the Australian government CI funding list and the National Disability Insurance Scheme list for CIs.

You are probably better off recruiting them from ENTs themselves who are saying to people, “right, let’s go. Let’s fit one ear.” And so, then you have got the people fresh, ready to go who are almost about to get surgery. (A3-F-I)

### Ethical considerations

#### Coercive reimbursement?

Most HCPs agreed that offering participants a CI is a generous and attractive form of reimbursement. However, several audiologists worried that, due to the large cost of a CI (approximately AUD$30,000 or £17,000GBP), reimbursement may be coercive, convincing either those who might not have previously considered a CI or those who may not need a CI to participate in the COACH study.

Maybe I am overreacting, but I see a big ethical issue in the design of the study in that … this dangling a cochlear implant to the hearing aid group at the end of the trial, like a bit of a carrot and saying it’s free, is almost coercive in terms of getting people to get a cochlear implant where previously they might not have thought about it or might not have considered that they needed one. (A4-F-I)

Other audiologists acknowledged the significant level of reimbursement but did not consider it coercive given the private and government funding available for cochlear implantation.

I wonder if people might be swayed because of the price tag that’s involved in fitting cochlear implants privately, though I understand a lot of it is fully subsidised these days or at least mostly, the costs are covered. (A5-F-I)

One audiologist was also troubled that Cochlear Ltd. might profit financially from RCT participants in the future.

If you are a cochlear implant manufacturer who is offering your product as a reimbursement to people to participate in the study, that product then needs to be serviced for the life of the product. And, if it breaks down or if it needs to be repaired or whatever, then you potentially gain. (A4-F-I)

#### Improving informed consent

Both HCPs and HA users flagged potential participants’ poor understanding or unrealistic expectations of CIs and suggested that the COACH study needs to ensure participants are fully informed of both the advantages and disadvantages of CIs.

You might get moderately severe people with hearing loss that do really well with hearing aids thinking ‘I'm going to get a cochlear implant out of it’. Thinking that a cochlear implant does X, Y and Z, because that's like the people that we get coming in who have heard about a cochlear implant and they think it's easier or better than hearing aids. (A2-F-I)

Because people only know what they’ve read or what’s on the internet or what they’ve been told. You know, they don’t know well enough, I guess, to be informed. Well, you want to make sure they are fully informed about you know, what if this doesn’t work out? What happens then? What if they can’t insert the electrodes as far as they want to? (A6-F-I)

I think it should be made clearer that the CI (due to the nature of the surgery) results in a hearing aid never being able to be worn again. Isn’t the risk meant to be that the CI may not work — despite the damage done to the cochlea? (HA7-E)

Similarly, audiologists suggested that participants should be told that taking part in the study would mean committing themselves to one of the three CI manufacturers. Others suggested that confidentiality should be an important component of the consent process and that participants’ details should not be distributed to any external parties (such as product marketeers). HCPs, HA users and support persons were also concerned about potential bias if Cochlear Ltd. were to run the study and recommended it be conducted independently of the company.

Are they making patients aware that there are two other brands of cochlear implants? Is that explained to them before they go through with it? Cochlear obviously is running the study, but they are also committing themselves to one manufacturer if they go ahead with it in the end. (A8-T-P)

And the first time one of these hearing clinics that we were part of the study sent information to us, marketing information … it has to be iron-clad. (HA3-SP3-F-P)

I think that it is probably a better way to go, the independent route, for people.

I mean, for audiologists, too, just to see that it’s not motivated by one manufacturer, just that they need their implants to get these results. (A7-T-P)

Some audiologists thought that sorting participants into the HA arm of the RCT after a lengthy and difficult decision-making process would be distressing for them. Conversely, some participants might find the transition from their existing HA to a new brand of HA challenging. HCPs suggest that these potential issues could be addressed by having counselling available from the randomisation period to the end of the study.

How do you deal with the disappointment? I know this happens, but you know, we’re talking a year away. That’s a huge you know, like we’re talking peoples’ emotions, peoples’ lives here. It’s just like, the quality of life. (A6-F-I)

Would they then be cheated out of the experience of what a cochlear implant might feel like, if they did two hearing aids for twelve months and then, they’re given the option to do the CI, would they have felt that those twelve months, I could have had more optimised through a CI? (A5-F-I)

People might have had [brand name] hearing aids their whole life and then, here you go, here’s [brand name]. They hate them. Just because they’re used to — you know, theirs … is there going to be sort of enough counselling? (A6-F-I)

### Refining trial conduct

Most HCPs, HA users and support persons could recognise the purpose of the study, acknowledge its importance, and thought that it was well designed. Several audiologists commented that the HA acclimatisation period was too short and that the study should allow for continuous adjustments in order to optimise HA performance. Audiologists suggested that the lead-in time needed to be more generous than 4 weeks and that additional consultations throughout the RCT would address hearing device discomfort, malfunction or any other issues.

Acclimatisation starts anywhere between three months after fitting hearing aids to six or even longer. Depends how many adjustments you need to make between first fitting to when the person’s adequately adjusted to wearing the hearing aids at their current setting. That time needs to be a bit more flexible. (A5-F-I)

A few months later they may feel they’re not hearing as well … or if they are not managing their device well and it’s quite blocked up with wax. (A7-T-P)

Many HCPs commented that the 2.5-h testing sessions would be exhausting or impractical for participants who were in employment, or could affect the study measures. One research audiologist recommended building breaks into the sessions, saying that would improve the COACH study from an ethical and a pragmatic point of view. Despite the length of the sessions, some audiologists thought that retention rates would not be impacted if participants were committed to the COACH study and were informed of the duration of the testing session at the outset of the COACH study.

I was involved in a research project where an appointment was three hours … we did build in two fifteen-minute breaks, just to go to the bathroom, or whether it’s to grab a cup of coffee, just to refresh and stretch your legs. But even so, we noticed a slight drop in performance in the latter part of testing, which was actually the harder bit of testing. I think ethics-wise would demand that of the researchers, the investigators build [breaks] in. (A5-F-I)

Two and a half hours seems to be a long time … And it depends if they’re working, you know, how old the people are. I mean, I guess you know that upfront. I think if people are committed, they will know ahead of time. (A6-F-I)

HA users provided mixed reports as to whether they could commit to five sessions, each lasting 2.5 h, over a year-long period. Some retired HA users and HA support persons stated that they had adequate time to participate in the study and that frequent hearing tests would be beneficial.

I’m not working, and I’m not tied up with anything else as such. So that would be okay. You know, if it helps anybody other than outside of me, that’s fine. And I do appreciate the technology advancement of all this, sort of, medical bits and pieces. (HA5-F-I)

Two and a half hours once a quarter is not an enormous ask, I don’t think … their hearing’s being continually checked for a year and it involves two and half hours, every three months, that’s not a great imposition. (SP1-F-I)

Other participants suggested that they would not be able to meet these time commitments due to their busy schedule or other practical concerns (such as travel time).

My husband’s visually impaired. I have to take him to – I don’t have to, but I do, take him to all his appointments plus my appointments, plus anything social for him, anything social for me. I just don’t want to get involved. (HA4-F-I)

I don’t like the idea of [husband’s name] driving on his own from home to here. So, no, I wouldn’t consider five times a year to come this distance. (SP4-F-P)

A small proportion of the HA users questioned what the study measured and what this actually meant, including what was involved. HA users were particularly curious about why quality-of-life tests were being administered. A few HA support people mentioned that their family members only used their HA sporadically and suggested that this behaviour could impact the study results.

Under study measures — not clear on whether you are aiming to establish a connection between communication and health-related quality of life and then back to hearing loss? (HA7-E)

It’s also, whether he’d keep the hearing aids in, because they were both hopeless … so, they’re just as likely to, just, operate with one, which then screws up your results, doesn’t it? (SP1-F-I)

Other HCPs wanted clarification about the extent of the reimbursement.

And rehab appointments. Like is it literally all free? Or is just the device? You know, ‘here is the implant but you have to pay for surgery’. (A3-F-I)

Some audiologists felt that the reimbursement would ensure retention rates over the 12-month period, while others felt that the participants should also be paid a small amount for every 2.5-h session in which they participated.

If you offer people $100 for even two and a half hours’ worth of testing, you will have no dramas with retaining people over the twelve months. (A3-F-I)

Consistent with earlier discussions about recruitment, HA users who expressed disinterest in CIs disagreed with the concept of randomisation, and would only agree to participate in the COACH study if they could be in the HA trial arm. Others refused to participate in the study outright, irrespective of whether it was nonrandomised and they were provided with a free pair of HAs. HA users that were more open to the idea of CIs stated that they would comply with the proposed randomisation.

I would just have to be as I am. I’m just a hearing aid person. (HA1-F-I)

I think I would participate, especially if it means that: A) I’m a suitable candidate and B) that I can be certain that it would improve my quality of life. (HA9-A5-F-I)

### Interconnected, accessible and appropriate methods of results dissemination

#### Responses to results

Most HCPs stated that the outcome of the study would be of interest to them, with many mentioning that it would influence their clinical practice.

A general statement about evidence or null hypothesis lack of evidence that cochlear implants in this cohort make a difference would be very useful. (GP5-F-P)

Obviously if the outcomes are less positive compared to what we have been led by being educated by cochlear implant companies and various places like that, then I would tell people to ‘really seriously consider it’. Obviously if it is extremely positive and people with implants do way better with the same hearing loss than two people with two hearing aids, then yeah, of course you would be really recommending it as an option for someone. (A3-F-I)

Some HCPs stated that the results of the COACH study would not change their practice because they rarely see patients with hearing loss or they frequently refer patients for CIs. One audiologist questioned how the COACH study is going to benefit the field, and what it will add to the knowledge base.

Like don’t we already know that a lot of people are going to say, ‘Well, I do better with my cochlear implant.’ And so what’s the point of the study? … I mean I see a lot of pre- and post-implant studies. (A4-F-I)

HA users had mixed feelings about the results of the COACH study, with some commenting that they would like to know the results so they “can make a comparison” (HA4-F-I), while others, particularly those who had little interest in using a CI, were preoccupied with other concerns (e.g. other comorbidities).

#### Tailored dissemination avenues

The main dissemination sites reported by HCPs were high-quality, peer-reviewed journals with their articles being accessed through a link in professional body publications (e.g. the *Audiology Australia* newsletter or how to treat via *Australian Doctor*) or via email from a colleague.

I don't sort of pull a journal out and just randomly — it has to be one that's targeted — so if somebody emailed me and said there's a journal article that pertains to this, I would then actively seek it. (A2-F-I)

During discussions with GPs about their role in relation to hearing loss and hearing devices as well as their clinical commitments, they clarified their reasons for their preferred dissemination methods.

GPs understand hearing loss. It's one of the things that's taught reasonably well in medical school and the physiology hasn't changed … It's a huge issue in general practice that there's often an assumption that we have deep knowledge on a whole range of issues … For issues like cochlear implants neither of us (gestures to the other GP in the paired interview) have a patient with a cochlear implant and actually is a waste of my mental hard drive to have lots of information on cochlear implants in … we’re better off seeking that information and making sure it's up-to-date if and when we need it. (GP5-F-P)

GPs expressed misgivings about receiving the results of the COACH study from Cochlear Ltd., because this source of information would require extensive assessment, a task they neither had the time nor the capacity to complete. Instead, GPs suggested that an unbiased medical expert should write a review of the COACH study in a trusted publication, frequently accessed by GPs.

Something like this coming from Cochlear, there's already a suspicion that it's a marketing exercise for Cochlear. Not the study itself … If it comes through as a report on a study that has shown … then we pick it up. (GP5-F-P)

A lot of the GPs read the medical newspapers and there’s a section called: ‘How To Treat’, and if you get one of the prominent ENT people who do a lot of the hearing stuff, then they write an article about hearing loss and then what’s up-and-coming section. (GP2-F-P)

Other HCPs emphasised the importance of seeing presentations about the COACH study at national conferences and being provided with links to journal articles.

I would expect something like this would be presented at a major conference to get that out to a lot of audiologists. Audiology Australia conference is the one I’m in. (A1-F-I)

Another significant suggestion was disseminating the results through the Cochlear Ltd. website within a professional resource tab.

That information might be accessible through a resources tab on the Cochlear webpage. Because if people hear cochlear, cochlear implants, they would literally search ‘cochlear’ and hopefully, that would come up … I think at least should be a one-pager and if it does get a peer review, then that article should be in the website, where it could be accessed. (A5-P9-F-I)

HA users wanted HCPs to inform them about the COACH study results either in a face-to-face manner or through written documents within the clinic. Face-to-face knowledge transfer would allow HA users to discuss the study and give them the opportunity to ask questions. Other HA users suggested that the GPs’ or audiologists’ waiting rooms would be an ideal place to advertise the study findings, either through pamphlets, posters, or on-screen. Some HA users recommended disseminating the information through hearing loss services like the *Deafness Forum of Australia* or *Better Hearing Australia*.

I would prefer to hear about any results regarding a study similar to the one in the diagram from a [doctor, audiologist, hearing impaired individual] who knows what they are talking about, is prepared to sit down with me and go through ALL the details of said study and its outcomes. (HA8-E)

Definitely have maybe a one-pager of a summary of the most crucial findings with a catchy title and leave it in the information brochures on the wall, or in GP or audiologists’ clinics. (HA9-A5-F-I)

## Discussion

This paper contributes to the limited body of evidence from embedded qualitative research in audiology trial studies [23], and helps to explore their design, conduct and dissemination of findings. The study adds new insights to the area of audiology research which could be expanded to other clinical trials. It identifies a range of important findings from user and service-provider perspectives that have implications for future research aimed at evaluating the effectiveness of hearing devices. q-COACH helps refine and enhance the feasibility and effectiveness of the COACH study, generating new ideas for inclusion in the design, improving recruitment and retention rates, informing consent procedures, conduct and delivery, and supporting the widespread dissemination of results. Importantly, it will ensure that the intervention meets the needs of HCPs, patients and support persons, adding to the body of pretrial qualitative research that has been shown to improve the quality of RCTs [25, 35, 36]. It will also help deliver vital information about the perceived value and benefits of data generated from the RCT and thus appropriate routes to intervention implementation [24]. If the results of the COACH study are to be effective, it is important that q-COACH informs the consultation approach of the RCT with patients and HCPs, meeting key stakeholder standards of acceptability and suitability, and that the information produced is disseminated to stakeholder groups in an appropriate manner.

The main barrier to recruitment and randomisation for COACH was the emergence of a clear treatment preference amongst patients. Most of the patients who were unsupportive of randomisation and study participation did so because they preferred HAs. The ability of these patients to choose their preferred hearing device meant that they were unwilling to relinquish control by accepting randomisation and, with it, the possibility of not receiving their preferred option. Conversely, those who accepted randomisation and participation in the COACH study were the participants who had expressed an interest in CI implantation. Patient preference for the type of hearing device was linked to their understandings and experiences of the different benefits and risks attached to each hearing device. Most saw the benefit of HAs as less invasive and performing adequately for everyday activities, while CIs were excluded as an option because patients feared surgery, considered themselves too old for surgery, were concerned that CI surgery was irreversible, feared losing residual hearing and found CIs aesthetically unappealing. The findings map to literature on both HCP perceptions of CI barriers and patient-associated barriers of CI uptake [33, 37, 38]. For instance, in a qualitative study of Australian and United Kingdom patients, Bierbaum et al. identified that patient concerns about CI surgery, potential loss of residual hearing and the irreversibility of the CI procedure were barriers to CI utilisation [33]. Our study demonstrates that these same barriers apply in relation to the recruitment and randomisation of participants in an RCT in audiology.

HCPs confirmed this finding by highlighting these barriers to CIs as well as the importance of clinical guidance and family support in countering these perceptions. Indeed, based on their clinical experience, HCPs highlighted that in cases where patients were strongly opposed to CIs it would be practically and ethically inappropriate to attempt to convince them to participate in the COACH study. Although few pretrial qualitative studies have been conducted in audiology, other qualitative studies have found that randomisation within RCTs is a major barrier to recruitment for patients with a clear treatment preference [35, 39, 40]. For instance, Harrop et al. found that most patients would not enrol in a bladder cancer trial because they saw the benefits of robotic surgery to be less invasive, requiring smaller cuts and producing less scarring, smaller risk of infection and shorter recovery period than for open surgery [41]. HCPs recommended that the COACH study tailor their recruitment avenues, targeting those who have already expressed an interest in CIs, are physically and psychologically prepared for CI implantation, and have adequate knowledge about the benefits and risks of CI implantation.

Other recruitment and consultation avenues arise when we consider patients’ treatment preferences as a more flexible and complex entity particularly when these preferences are based on incomplete or inaccurate information. Mills et al. [42] argue for the importance of training the recruiter to engage with preferences, which often leads to preferences dissipating and the patient making a more informed decision that often includes randomisation. Given this, it may be beneficial for the COACH study to train recruiters to engage with patient preferences including discussing reasoning and correcting accuracies, rather than accept them at face value. Exploring treatment options can allow patients the opportunity to fully consider all treatments and trial participation, which can improve recruitment rates and facilitate informed consent [42].

A diverse portfolio of recruitment sites within Australia was recommended to attract participants who were both psychologically and physically prepared to embark on cochlear implantation. As other literature suggests [43], RCTs should consider multiple approaches to maximize recruitment rates, which can be improved by inviting people to face-to-face sessions. For instance, eligible patients who routinely attend audiology or ENT clinics could be identified and invited to face-to-face sessions which may improve the recruitment rate.

q-COACH identified several ethical quandaries including the notion of an incentive, which could be seen as coercive if recruitment is not tailored appropriately, and more comprehensive consent procedures to offer a more informed understanding of the study and better opportunities for user-support structures which could be built in to the COACH design. Participants concern over the potentially coercive nature of the CI illustrates the importance of a strict eligibility criterion prior to acceptance into the COACH study which includes both surgical screening and appropriate imaging. Other studies have highlighted emotional challenges of trial work [44, 45] such as the emotional labour that staff are expected to undertake and strategies they are expected to employ to pre-empt or manage patients’ responses to randomisation results [46]. We recommend, as others have [46], that participants and staff receive practical, emotional and specialist support, such as having access to a psychologist or counsellor, role play to help staff develop strategies for addressing patients’ emotional responses, and opportunities to share good practice amongst staff. Moreover, counselling staff should be available during all phases of the trial to ensure that ethical issues are addressed properly including assessment of the benefits and risks of cochlear implantation and awareness of CI brand choices prior to the randomisation phase. The ethical problems highlighted in q-COACH will improve informed consent and ensure COACH supports its staff and is adequately prepared for participant involvement.

Many participants suggested that the length, content and availability of the study measure sessions proposed should be altered or more clearly explained to accommodate participants’ needs, daily lives (including work schedules) or existing knowledge base. The everyday habits of HA users were also flagged as a possible impediment to the study, and more detail about the extent of reimbursement is required. The available literature supports this finding, suggesting that 24% of the people given an HA do not use them [47]. The main barriers to the consistent use of HAs, as reported by patients, are often related to poor performance, poor fit, and discomfort [48]. Therefore, it is crucial, as our study indicates, that regular adjustments are possible to ensure that they perform well. Moreover, the COACH study also needs to accurately capture HA usage data so that any potentially confounding impact on results can be detected.

Some audiologists felt that the main form of reimbursement (the CI) would help retain participants in the study over the 12-month period, while others felt that the participants should be paid a small amount for every 2.5-h session in which they participated. As discussed above, and consistent with the existing literature on randomisation in other fields [35, 39–41], potential participants who had an opinion of a preferred treatment disliked the concept of randomisation and said they would not participate in the study. HA users who were open to the idea of a CI, however, stated that they did not mind being randomly allocated to one or other trial arm. This finding is novel in the field of audiology, and the recruitment strategy suggested (targeting those who have expressed an interest in CIs) will be of use for both the COACH study and future RCTs conducted in audiology.

Whilst initial patient preferences to the intervention might reasonably influence subjective outcomes, this would not be expected to alter objective outcomes (for example, results on an audiogram). Furthermore, such outcome measures may be analysed in a single-blind fashion with the assessor blinded to treatment allocation. Given that patient expectations or preferences may mediate subjective outcomes, some record of initial preference might be warranted to enable the researchers to examine the influence of these prior preferences on results.

Despite the importance of information translation to increase the awareness of new knowledge and achieve change in audiences’ actions or behaviours, pretrial qualitative research rarely seeks stakeholders’ perspectives on dissemination strategies for RCT results. Adequate information in dissemination is of particular importance in audiology where hearing device knowledge amongst HCPs and patients is insufficient or compromised [13, 17, 18, 37, 38] and the uptake of both HAs and CIs is low [8–10]. Cohen et al. [17], for example, found poor awareness of, and little knowledge about, CI eligibility criteria amongst primary care providers in the United States and recommended increased training and outreach by CI specialists. Australian GPs also acknowledged that they lack confidence and knowledge about CIs and about CI candidate eligibility criteria to provide adequate counselling on this and on referral. This in turn has led to fewer GP referrals than might be expected with this patient cohort [33].

q-COACH reported that COACH should be published in a high-quality, peer-reviewed journal with a summary of the research and a link to the relevant journal article made available through existing professional resources and networks (for example, professional newsletters or colleagues). Specifically, GPs commented that they have a sophisticated understanding of hearing loss, and for the purposes of their everyday clinical practice only required a brief review of the COACH results by an unbiased medical expert (such as an ENT surgeon) within a specialist-specific publication. Given the concern regarding bias, we recommend that the study itself and its dissemination methods are free of any conflict of interest. For example, Cochlear Ltd. should not be involved in study execution, monitoring or reporting and there should be a commitment to publish all results. This commitment should be independent from funding.

Other HCPs suggested receiving information through conference presentations or via the Cochlear Ltd. website. HA users and support persons preferred that HCPs inform them of the study either face-to-face or through visual or written materials in clinical waiting rooms. q-COACH therefore provides valuable insight into the important features of information sources including the perceived credibility with, and proximity to, the target stakeholder groups, the level of clarity and brevity of the content, and the appropriate channel for each target audience [49]. As Marriot et al. [49] remind us, effective information dissemination involves multiple stakeholders being approached through various means, each receiving tailored information for their task and information needs. Equally, we must be cautious not to overburden stakeholders with information they will not, or cannot, use [49].

We argue that pretrial qualitative studies like q-COACH are of most use when they allow for an understanding of stakeholders’ experiences, roles, and comprehension of the phenomenon in question (in this case, hearing loss and hearing devices). This will enable information dissemination needs to be considered within the context of each stakeholder cohort. By speaking to a wide range of stakeholders, q-COACH also illustrates how dissemination methods are interconnected and interdependent. An understanding of the methods of dissemination, the features of information sources and how they are linked is essential if we wish to support the transfer of knowledge from the COACH study into the real world. Knowledge translation of COACH has the potential to improve clinical practice, referral pathways, and patients’ quality of life through the obtaining of the most appropriate hearing device.

### Limitations

The first limitation is the use of snowball sampling which can generate a community bias. For example, the majority of audiologists were recruited from only two Sydney-based clinics. We can speculate that if we had interviewed more audiologists across Australia we may have generated a richer and more complex picture of the phenomena. The second limitation is the hypothetical nature of the study to determine participation barriers. Despite this limitation, pretrial qualitative work was necessary in the audiology field where engagement with stakeholders is rare.

## Conclusion

q-COACH has key implications for the COACH study, identifying both benefits and challenges to its design, conduct and dissemination of results amongst stakeholders. This pretrial study has also provided recommendations for RCT intervention and trial delivery in general, including the dissemination of results which can be executed in advance of a trial commencing, thus pre-empting problems relating to trial acceptability and feasibility. More broadly, this study provides insights into RCTs in the field of audiology, where pretrial qualitative research is rarely undertaken, and therefore findings may be transferrable to other RCTs in audiology.

q-COACH has also highlighted the value of conducting qualitative research with multiple stakeholders to better understand health conditions and clinical care, while offering opinions on proposed RCTs. q-COACH has revealed how important it is that COACH implementation and design is in consultation with key stakeholders to ensure its acceptability and suitability [50] and that this information is disseminated in an appropriate manner [51]. This has clear implications for the design of future pretrial qualitative research, aiming at increasing the value and impact of qualitative research in relation to RCTs.

## Data Availability

The datasets generated and/or analysed during the current study are not publicly available due to the nature of the consent provided by participants, but anonymised data are available from the corresponding author on reasonable request.

## Abbreviations

CI: Cochlear implant
COACH: Comparison of outcomes with hearing aids and cochlear implants in adults with moderately severe-to-profound bilateral sensorineural hearing loss
ENT: Ear, nose and throat
GP: General practitioner
HA: Hearing aid
HCP: Healthcare professional
q-COACH: The qualitative Comparison of outcomes with hearing aids and cochlear implants in adults with moderately severe-to-profound bilateral sensorineural hearing loss study
RCT: Randomised controlled trial
